# Pennsylvania State Veterinary Medical Association

**Published:** 1901-06

**Authors:** 


### SOCIETY PROCEEDINGS.

### PENNSYLVANIA STATE VETERINARY MEDICAL ASSOCIATION.

(Continued from page 339.)

The following are the officers and committees for 1901–1902:

*President*—S. J. J. Harger, 205 N. Twentieth Street, Philadelphia, Pa. *First Vice-President*—W. L. Rhoads, Lansdowne, Pa. *Second Vice-President*—M. Moriarty, Gettysburg, Pa. *Third Vice-President*—C. W. Boyd, Pittsburg, Pa. *Treasurer*—Francis Bridge, Philadelphia, Pa. *Recording Secretary*—C. J. Marshall, 2004 Pine Street, Philadelphia, Pa. *Corresponding Secretary*—E. M. Ranck, 422 N. Forty-first Street, Philadelphia, Pa.

*Committee on Legislation*—M. E. Conard, West Grove, Pa., *Chairman;* J. W. Sallade, Pottsville, Pa.; J. C. McNeil, Pittsburg, Pa.; Otto Noack, Reading, Pa.; Benjamin Underhill, Media, Pa.; W. Horace Hoskins, Philadelphia, Pa.; F. F. Hoffman, Brookville, Pa.

*Committee on Intelligence and Education*—Jacob Helmer, Scranton, Pa., *Chairman;* J. W. Adams, Philadelphia, Pa.; George Magee, Uniontown, Pa.; B. F. Senseman, Philadelphia, Pa.; H. P. Keely, Schwenksville, Pa.; C. Goentner, Bryn Mawr, Pa.; J. Butterfield, South Montrose, Pa.

*Committee on Sanitary Science and Police*—C. C. McLean, Meadville, Pa., *Chairman;* R. G. Rice, Towanda, Pa.; J. M. Courtright, Clark's Green, Pa.; H. S. Jackson, Sewickley, Pa.; Charles Williams, Philadelphia, Pa.; C. J. Marshall, Philadelphia, Pa.; J. B. Irons, Erie, Pa.

*Committee on Animal Husbandry*—George B. Jobson, Franklin, Pa., *Chairman;* W. H. Ridge, Trevose, Pa.; A. W. Radley, Bethlehem, Pa.; Leonard Pearson, Philadelphia, Pa.; J. C. Michener, Colmar, Pa.; E. C. Porter, New Castle, Pa.; W. H. Fry, Pine Grove Mills, Pa.

*Trustees*—Leonard Pearson, *Chairman*, Philadelphia, Pa.; W. Horace Hoskins, Philadelphia, Pa.; H. B. Felton, Olney, Philadelphia, Pa.; Thomas B. Rayner, Chestnut Hill, Pa.; N. Rectenwald, Pittsburg, Pa.

For the New Jersey Veterinary Medical Association President Herbert A. Lowe, of Paterson, was called to report. Dr. Lowe spoke of the harmonious workings of the amalgamation of the three societies in that State. He also spoke of the untiring efforts of those who were influential in bringing about this union, and said that the fire was kindled in our society when Dr. Hoskins criticised the chaotic condition of the societies in his State. Immediately he went to work to unite these societies into one State organization, which was accomplished as a closing triumph of the nineteenth century. He was rewarded for his efforts by being chosen the first president.

Dr. Hoskins was called to report as a delegate to the New York Veterinary Medical Association. As he was in attendance at the American Veterinary Medical Association at this time, he could not respond.

Dr. Marshall reported the Keystone Veterinary Medical Association in a flourishing condition, but thinks there should be a larger attendance especially of the city veterinarians. Of the seventy-six veterinarians registered in Philadelphia, about twenty are members of this Society, and about half that number active members, but what it lacks in quantity is made up in quality. Those who attend feel well repaid for their efforts.

Dr. Pearson called for a report from the Schuylkill Valley Society. Drs. Noack and Schneider reported this society in good condition and good prospects for improvement.

Dr. Hoskins spoke of the sad loss to the Maryland Society in the death of Dr. A. W. Clement, which occurred in Baltimore last Sunday morning. He was one of our most sturdy workers for the benefit of the profession in his city, State, and nation.

The Treasurer's report was made by Dr. Bridge, which was accepted and filed. (Will appear elsewhere.)

*Reports of Committees.*

Owing to the absence of Dr. M. E. Conard, chairman of the Committee on Legislation, the report of this committee was postponed. Dr. Conard was in Harrisburg opposing the Styles bill that had passed the Senate, and proposed to reopen the registration act of 1889.

*Committee on Intelligence and Education.*—The chairman of this committee, Dr. H. B. Felton, read his report, which was very interesting and instructive. His report was arranged in a clear and concise manner, which is characteristic of Dr. Felton, and filed with the secretary. A vote of thanks was extended to Dr. Felton for his excellent report.

*Committee on Legislation.*—The Committee on Legislation was next called. Dr. W. H. Ridge reported that he had sent out 1762 letters in reference to the recent Army bill. Dr. Jobson was called but had no report to make. Dr. Hoskins responded and spoke of the Styles bill which had passed the Senate. This bill was introduced by Senator Styles, of Allentown, for the purpose of allowing a few men to register who neglected to do so in 1889. He considered this bill badly worded, as it allows anyone to register who assumes the title of veterinarian. It would make our profession the dumping-ground for all the quacks and impostors in our own and adjoining States. He spoke of the necessity of killing this bill in the committee if possible. He outlined the work necessary to kill it in the House. Drs. Harger and Sallade also spoke of the necessity of prompt and united action in this matter.

At 1.30 P.M. the meeting adjourned for dinner at the University of Pennsylvania restaurant.

AFTERNOON SESSION.

The afternoon session was called at 2.30 by President Harger. A photograph of the Society was taken in the lecture-room. Dr. Conard was then called to make his report as the chairman of the Committee of Legislation. He made a verbal report, and emphasized the necessity of looking after the Styles bill at Harrisburg.

Under the subject of new business, Dr. E. M. Ranck spoke of the necessity of supplying money for the Board of Examiners for the prosecution of illegal practitioners. He thought it would be advisable to appropriate some money from this Society to be used by the board at its own discretion. President Harger said that the money used at present for this purpose was supplied by the individual members of the board, and owing to the fact that they gave their services gratuitously, the money expended for this purpose should be raised in some other way. This board was originated by our Society, and he agreed with Dr. Ranck that the parent society should contribute a portion of the necessary expenses.

Dr. Otto Noack said he thought it would be an unsatisfactory way to dispose of the funds of the Society. He sees but little satisfaction in prosecuting even if the person prosecuted is convicted. One man in his county was prosecuted and convicted, but afterward appointed by the American Veterinary Society as county secretary during the recent efforts for the passage of the Army bill. There are men of good professional standing in his county who would have been pleased to serve in this capacity, and he thinks it an imposition for this Society to so honor a man who had been convicted of illegal practice. W. H. Ridge explained how this circumstance occurred. He sent a list of all the veterinarians in this State to Dr. Huidekoper at Washington. Many of these men were not even registered. Dr. Huidekoper realized the necessity of numbers in this undertaking and chose these county secretaries more especally from a geographical point of view, and having been absent from the State for some time, had probably overlooked the matter.

Dr. W. S. Kooker moved that this Association donate $200 for the purpose of prosecuting illegal practitioners, and that the board be authorized to use this money or any part of it at its own discretion. This motion was seconded. Dr. Rhoads was opposed to this motion, and does not consider this Association able to contribute that much money. That $200 would rob our Association, and only be a drop in the bucket for the examining board, and the Association could spend its money for other purposes of far more importance.

Dr. Deaver was not in favor of spending the money of the Association unless in cases of emergency, because without money it would lose its prestige and influence.

Dr. Kooker cited several cases that had been prosecuted, and for the lack of money no satisfaction had been obtained. In most of these cases the magistrate favored the one prosecuted, and he feels sure that had the board had means to carry the case to a higher authority the decision of the magistrate would have been reversed. He thinks we should make a test case and carry it to the Supreme Court if necessary to establish a precedent.

Dr. E. E. Tower was opposed to the motion, and he thought this amount of money could be used a great deal more profitably at present in opposing the Styles bill. Dr. George Jobson agreed with Dr. Tower that this would be a more sensible way of using the money. Dr. Noack attempted to offer a motion to this effect, but President Harger would not entertain it, as there was already a motion before the house. Dr. J. B. Irons thought that an honest decision could be obtained in his county in case of a prosecution.

Dr. Rhoads said the expense of a prosecution should be borne by the man or men who would be benefited by the conviction. This would tend to make a man positive that he had evidence enough to convict before making complaint. He would prefer that the money be raised by subscription rather than to be taken from the funds of the Association.

Dr. Bridge gave the financial condition of the Association at present. Last year $114 was spent in the necessary expenses of running the Association. Rather than appropriate $200 at one time for this purpose, to be used indiscriminately, he would be in favor of appropriating such an amount as the Association deem wise when it is known for what purpose it is to be used. The motion was put and lost.

E. M. Ranck suggested that a committee on Animal Husbandry should be appointed, and also read a letter from Dr. Huidekoper which was received and filed.

Dr. Kooker asked for an explanation and the object of the Committee on Animal Husbandry. Drs. Ranck and Harger explained the necessity for such a committee.

Dr. Herbert A. Lowe said that the New Jersey Association had a similar committee, which had been found to be very useful and beneficial.

Dr. Kooker moved that a Committee of Animal Husbandry be appointed, consisting of five members. Rhoads thought there should be six, and Ridge wanted seven men appointed. After the motion was amended to have seven members the question was put to a vote. The yeas and nays were called, twenty voting in the affirmative, fourteen in the negative. Dr. Rhoads moved that the work be outlined for this committee by the incoming Board of Trustees. This motion was seconded and unanimously adopted.

As the American Veterinary Medical Association is to hold its next meeting at Atlantic City in September, Dr. Ranck urged the necessity of the individual and united efforts of the veterinarians of this State in assisting to make that meeting a success.

Dr. Lowe responded, and complimented the veterinarians of our State and Association for the assistance already given in aiding them to secure this meeting of the National Association. We are attempting to make this one of the best national meetings ever held, and feel confident of your support. Dr. Noack suggested the feasibility of holding our semi-annual meeting at the time of the national meeting in Atlantic City. The subject was not in order at the present time. Dr. Lowe said Dr. Hoskins had been appointed by the President of the Association to look after the local arrangement. My State has also appointed five members as a committee on local arrangements. Dr. J. T. Ferley made a motion that the president appoint five members to act on a similar committee. The motion was seconded and carried.

Dr. Ranck read a report of a special meeting of a few members of this Association, which was held for discussing arrangements for the meeting at Atlantic City. Dr. Harger explained that this committee was only in vogue until the annual meeting of our Association. Dr. Mahaffy offered some suggestions for the local arrangements at the national meeting which Dr. Schreiber thought were nice but not practical.

President Harger spoke of the deaths of Drs. Reinhardt, Clement, and Jonathan Price, two of whom were active members in our Society. Dr. Ranck moved that a committee of three be appointed by the president to draw up suitable resolutions on the death of these men. The motion was regularly adopted. The president appointed on this committee Drs. Pearson, Noack, and Magee.

As visitors from the Veterinary Medical Association of New Jersey, the

President, W. Herbert Lowe, H. W. Read, L. P. Hurley, Charles E. Magill, James M. Mecray, and A. F. Sellers were called, and each made a few remarks.

Dr. Jacob Helmer, of Scranton, as chairman on Sanitary Science and Police, presented his report. It was accepted and filed.

Dr. J. B. Irons presented a report of the committee appointed at Erie on the Registration of Stallions, and spoke of a law that was passed a few years ago requiring the owners of stallions to have them registered with the prothonotary in each county. He thinks there should be an amendment recommended to this law requiring a certificate of soundness before the horse is eligible for registration. In this county there is a book kept for the registration of stallions and about a dozen are registered, but as there is nobody to look after this matter the requirements are not enforced. Dr. Butterfield explained that as a penalty for not registering a stallion the fee for his services should not be collected. Dr. Hoskins thought a committee of five should be appointed to look after this matter, with J. B. Irons as chairman. He made a motion to that effect, which was seconded and adopted unanimously.

*Reading of Essays and Papers.*

Dr. J. B. Irons, of Erie, Pa., read a paper on "Pericarditis." Dr. Bridge opened the discussion and described cases that have come under his observation. Dr. Pearson described a few cases and spoke of the difficulty of making a positive diagnosis. Some of the most predominant symptoms, he considered, were œdema of the dewlap, pain elicited on movement, tender over the point of the xiphoid cartilage and over the region of the second stomach, loss of appetite, colicky pains and sluggishness of the animal, sometimes there is a hacking cough and usually rapid, shallow breathing, animal does not lie down, painful defecation. With this class of symptoms there is but little difficulty in making a diagnosis. There is a possibility of its being confused with tuberculosis.

The question was opened for general discussion, and Dr. Ridge cited a case in which he had made a diagnosis of tuberculosis. This happened several years ago, when the subject of tuberculosis began to be agitated. Thinking the case would be interesting for study at the hospital, it was taken from Holmesburg to the University of Pennsylvania in an ambulance. The autopsy revealed traumatic pericarditis caused by a piece of telegraph wire and no indications of tuberculosis. Dr. Michener, having had considerable experience in treating this disease, gave as the most diagnostic symptoms a splashing sound on auscultating the heart, indigestion, and swelling of the dewlap. Dr. Rectenwald reported a few cases which had made a satisfactory recovery from this trouble. One case in which a darning-needle worked through the pectoral muscles formed an abscess, and was removed. In another case a piece of hay wire was removed in the same way. One case which he was called to see the owner thought had been bewitched, but on autopsy a shawl-pin was found to account for the mysterious actions of the animal. Among the symptoms he considers swelling of the dewlap most positive.

Dr. Bradley inquired if horses were subject to pericarditis. Dr. Pearson explained that traumatic pericarditis was very unusual in a horse, but that they do sometimes have pericarditis as a secondary disease. The symptoms are great depression, increased area of dulness on percussion, heart-beat rapid and weak, pulse irregular and thready, animal very weak and may faint, due to lack of blood in the vessels, loss of appetite, twitching of the muscles, trembling, respiration rapid and temperature high. The prognosis is bad. Dr. Jobson spoke of a horse that had heaves and died. Pericarditis was found on autopsy.

The paper on "The Treatment of Parturient Apoplexy," by E. M. Michener, of North Wales, was read by his father, J. C. Michener, of Colmar, and will be published in the June number of the JOURNAL.

On motion, the meeting adjourned until the morning of March 6th.

### SECOND DAY'S SESSION.

On March 6th, Dr. S. J. J. Harger, the President, called the meeting to order at 10.26 A.M.

The Secretary, Dr. Marshall, read a paper for Dr. B. F. Underhill entitled "Spare Hours." The paper dealt with the use to be made of hours not employed by the absolute demands of practice.

Dr. W. H. Ridge presented a paper on "Veterinarians' Interest in Agricultural Education." (The paper will appear later in this JOURNAL.)

Communications were read from F. F. Hoffman, Brookville, Pa.; Dr. Kingsland, Canton, Pa., and Dr. Claude D. Morris, Secretary of the New York State Veterinary Medical Society.

Dr. Pearson called attention to a collection of instruments and books. He stated that these were the property of Dr. Reinhart, a member of this Association for a great many years, who died a short time ago. Mrs. Reinhart wished to have these effects disposed of in some way and placed this matter in the hands of Dr. Sallade and himself to dispose of them the best way they could, and that they had thought it the best plan to get them down for this meeting so all could see them, and to sell them or auction them off and realize as much as possible on them for her benefit. He hoped something of that sort could be done.

The President authorized an auction to be held.

Dr. Jacob Helmer read a paper on "The Problem of Municipal Milk-inspection." (See page 205, April number of this JOURNAL.)

Dr. Hoskins: I learned this morning that our colleague, Dr. Clement, of Baltimore, will be buried at 1.30 at Lawrence, Mass. I telegraphed Dr. F. Winchester asking him to place upon the body some flowers as a token of esteem from the Pennsylvania veterinarians.

The meeting adjourned at 12.45 P.M. for lunch.

### AFTERNOON SESSION—*March 6th.*

The meeting was called to order by the President at 3.05 P.M.

The consideration of Dr. Helmer's paper on "The Problem of Municipal Milk-inspection" was taken up. The discussion was opened by Dr. C. J. Marshall, and proved valuable and interesting. (See page 218, April number of the JOURNAL.)

The President noticed the entrance of one of the honorary members, Dr. Rush S. Huidekoper, and invited him to the amphitheatre, requesting a few words.

Dr. Huidekoper said: It is a great pleasure to be with you all. I remember the time when if we got twelve or fifteen men together we thought we were having a good meeting, and it is a great pleasure to see the meeting grown to an attendance of nearly two hundred, but the programme is too interesting for me to interrupt you.

Dr. Charles Williams made an address on "Foot-sore Horses in Our City and Suburbs," with a view to ameliorating and preventing the same. (The paper will be published in a later number of the JOURNAL.)

Dr. J. W. Adams gave a paper on "Neurectomy in the Treatment of Spavin," illustrated by dissected specimens and life-size drawings. (The paper will be published later in the JOURNAL.)

The Board of Trustees reported favorably upon two applications for membership—Dr. Harry T. McNeil and Dr. Cornan.

The President said he took great pleasure in introducing Dr. E. L. Cornan and Dr. Harry T. McNeil.

Dr. Pearson stated: Mr. President, I have the report of the Secretary for the year that closed when this meeting opened. It is accompanied by vouchers covering the printing bills. The report shows that the Secretary has taken in during the year $82; that he has expended for postage, printing, and expressage 83 cents, altogether $65.99, leaving a balance of $16.31 that has been turned over to the Treasurer. The report has been carefully gone over and found correct.

It was moved and seconded that the report be accepted.

Dr. Pearson offered a resolution on the death of Dr. Reinhart. On motion that it be accepted, it was duly seconded and carried, and a motion that a copy of these resolutions be sent to the family was duly seconded and carried.

Dr. Pearson also read a resolution relative to Claude R. Morris' expulsion from membership. Upon motion, duly seconded, this resolution was adopted and carried.

Dr. Sallade offered a resolution in regard to the death of Dr. A. W. Clement. Upon motion, duly seconded, the resolution was adopted and carried.

Dr. Sallade offered a second resolution. Upon motion being duly seconded the same was adopted and carried.

Dr. Sallade offered a third resolution *in re* animals bitten by rabid dogs. Upon motion to adopt being duly seconded the same was carried.

Dr. Sallade offered a fourth resolution *in re* the suppression of the diseases of animals. Upon motion to adopt being duly seconded the same was carried.

Dr. Jobson offered a resolution *in re* quarantine of rabies. Upon motion to adopt being duly seconded it was carried.

Dr. Jobson offered a second resolution *in re* new veterinary school. Upon motion to adopt being duly seconded the same was carried.

Dr. Jobson offered a third resolution *in re* sanitary control methods, supplemented by commendatory remarks upon Dr. Pearson's work. Upon motion to adopt being duly seconded the same was carried, with a supplemental motion that a copy of the resolution be sent to Dr. Pearson, which was likewise carried.

Dr. Pearson offered a resolution *in re* milk and meat supply. On motion to adopt being duly seconded the same was carried.

[The above resolutions appeared on pages 266, 267, and 268, April number of the JOURNAL.]

Dr. Pearson said: Mr. Chairman, I have just read a telegram from Dr. Conrad, and I have just come from a conversation with him over the telephone in regard to the Styles bill. The hearing on this bill, to let down the bars and permit anyone to register, will be held to-morrow evening at 7.30 in the committee room—Committee on Public Health and Sanitation—which is in room 153. He says there is a disposition to rush this bill. Senator Styles had been to see the members of this committee and had urged them to get it out at once, and it was only with great difficulty that it was held back until to-morrow night. The chairman of the committee said that he did not think that it could be deferred longer than to-morrow night. It seems to me that it is important that we should be heard before that committee, and I think it highly important that we should be heard in an impressive way. There is strength in numbers, and if there is a large body of men going to Harrisburg to-morrow on the train leaving here at 4.50, arriving there at 7.15, they could go at once to the committee room, be heard, and get away from Harrisburg at 1 o'clock, and get down here Friday morning. I think it might be the means of saving this measure. I would suggest that everyone that can prepare to go to Harrisburg to-morrow afternoon, so we will know what can be counted on, and notify Dr. Conrad, who is chairman of the Legislative Committee. I would like to ask those to rise who can do this.

The President: The proposition is, all those who think they can go to Harrisburg to-morrow and do whatever they can to prevent the passage of this bill will please rise; those who think they are able to go and work against the bill.

Dr. Pearson: Mr. Chairman, I think this is a very vital matter, and I think everyone should strain a great point to go to Harrisburg to-morrow. Everyone's presence counts as a very great factor, and it seems to me that I would be willing to give up everything for a couple of weeks in order to be present to-morrow night at this committee meeting. It is now or never.

The President: It is now or never.

Dr. Pearson: It is not a matter of a couple of weeks, it is a matter of years.

The President: All those who are willing to go please rise. (Secretary Ranck counts about twenty.) We would be pleased to have all who can possibly go.

Dr. Pearson: Dr. Rhoads makes a very important suggestion. He says if we can be sure of twenty-five men we can have a special car up and a special car back. It may be difficult to get back on that 1 o'clock train without some special provision.

A Member: Dr. Pearson, there are quite a number of those twenty who go West.

Dr. Moriarity: All those gentlemen who cannot go send a telegram to the representative of their county. It only costs a quarter, and will do us some good to send them a telegram urging them to vote against the bill.

(To be concluded.)

---

The National State Association of Dairy and Food Commissioners will convene in October at the Pan-American Exposition, Buffalo, N. Y.

